# The New 3D Printed Left Atrial Appendage Closure with a Novel Holdfast Device: A Pre-Clinical Feasibility Animal Study

**DOI:** 10.1371/journal.pone.0154559

**Published:** 2016-05-24

**Authors:** M. Brzeziński, K. Bury, L. Dąbrowski, P. Holak, A. Sejda, M. Pawlak, D. Jagielak, Z. Adamiak, J. Rogowski

**Affiliations:** 1 Department of Cardiac and Vascular Surgery, Medical University of Gdansk, Gdansk, Poland; 2 Faculty of Mechanical Engineering, Gdansk University of Technology, Gdansk, Poland; 3 Department of Surgery and Roentgenology, Faculty of Veterinary Medicine, University of Warmia and Mazury Olsztyn, Olsztyn, Poland; 4 Department of Pathomorphology, Medical University of Gdansk, Gdansk, Poland; Medical University of Gdańsk, POLAND

## Abstract

**Introduction:**

Many patients undergoing cardiac surgery have risk factors for both atrial fibrillation (AF) and stroke. The left atrial appendage (LAA) is the primary site for thrombi formation. The most severe complication of emboli derived from LAA is stroke, which is associated with a 12-month mortality rate of 38% and a 12-month recurrence rate of 17%. The most common form of treatment for atrial fibrillation and stroke prevention is the pharmacological therapy with anticoagulants. Nonetheless this form of therapy is associated with high risk of major bleeding. Therefore LAA occlusion devices should be tested for their ability to reduce future cerebral ischemic events in patients with high-risk of haemorrhage.

**Aim:**

The aim of this study was to evaluate the safety and feasibility of a novel left atrial appendage exclusion device with a minimally invasive introducer in a swine model.

**Materials and Methods:**

A completely novel LAA device, which is composed of two tubes connected together using a specially created bail, was designed using finite element modelling (FEM) to obtain an optimal support force of 36 N at the closure line. The monolithic form of the occluder was obtained by using additive manufacturing of granular PA2200 powder with the technology of selective laser sintering (SLS). Fifteen swine were included in the feasibility tests, with 10 animals undergoing fourteen days of follow-up and 5 animals undergoing long-term observation of 3 months. For one animal, the follow-up was further prolonged to 6 months. The device was placed via minithoracotomy. After the observation period, all of the animals were euthanized, and their hearts were tested for LAA closure and local inflammatory and tissue response.

**Results:**

After the defined observation period, all fifteen hearts were explanted. In all cases the full closure of the LAA was achieved. The macroscopic and microscopic evaluation of the explanted hearts showed that all devices were securely integrated in the surrounding tissues. No pericarditis or macroscopic signs of inflammation at the site of the device were found. All pigs were in good condition with normal weight gain and no other clinical symptoms.

**Conclusion:**

This novel 3D printed left atrial appendage closure technique with a novel holdfast device was proven to be safe and feasible in all pigs. A benign healing process without inflammation and damage to the surrounding structures or evidence of new thrombi formation was observed. Moreover, the uncomplicated survival and full LAA exclusion in all animals demonstrate the efficacy of this novel and relatively cheap device. Further clinical evaluation and implementation studies should be performed to introduce this new technology into clinical practice.

## Background

Atrial fibrillation (AF) is the most common cardiac arrhythmia, affecting in total 2.5 million people in the United States[1] and 6 million people in Europe, and these numbers are expected to double in the next 50 years.[2] It is estimated that AF occurs in every tenth patient over 80 years of age. The main complications of AF are thromboembolism and stroke.[3] One in five ischemic cerebral incidents is associated with cardiac arrhythmia, and the highest mortality rate is associated with those incidents caused by atrial fibrillation. Patients who survive such incidents more often remain incapacitated and are often exposed to repeated episodes of stroke. As a result, in patients experiencing ischemic cerebral incidents caused by atrial fibrillation, the risk of death is two-fold greater and the cost of treatment is 1.5-fold higher.[4] Furthermore, most commonly, AF develops without symptoms; therefore, stroke can be the first sign of AF.

The most common form of treatment for atrial fibrillation and stroke prevention is pharmacological therapy with anticoagulants. [5–8] Studies have shown that anticoagulation therapy with warfarin and dabigatran as well as new oral anticoagulants (apixaban, rivaroxaban and edoxaban) decreases the incidence of stroke in patients with atrial fibrillation, nevertheless those drugs are associated with a high risk of major haemorrhage as high as 4%.[9,10] Therefore, the search for better prevention methods should be focused on non-pharmacological interventions for preventing the formation of clots. [4,5,11] The new intervention that has gained the highest popularity is left atrial appendage (LAA) occlusion.

More than 90% of atrial clots in non-rheumatic atrial fibrillation cases form in the LAA[5]; therefore, LAA exclusion can reduce the incidence of clot formation and, thus, the risk of stroke. Currently, many researchers are attempting to develop solutions to LAA closure that allow it to be quick, effective and minimally invasive. As a result, several patent applications have been submitted, but few studies have been performed. [2,12,13] None of the recently developed devices has shown clear superiority. Percutaneous instruments as well as suture techniques and staples have resulted in atrial injuries and bleeding or incomplete occlusion of the LAA with residual flow and residual thrombus.[7] A more recent study by Kanderian and colleagues compared the success rate of surgical excision of the LAA with suture or stapler exclusion in 137 patients undergoing open-heart surgery. They have reported a higher rate of successful closure with excision (73%) than with suture exclusion (23%) or stapler exclusion (0%).[8] However, the methods that have been proposed recently are expensive and, more importantly, do not guarantee success or ease of application. Thus, it is highly important to improve this strategy or propose a new method that is not only effective but also easy to apply with low cost.

The aim of this study was to assess the safety and feasibility of a newly developed occlusion device in vivo and to investigate its utility for occluding swine atrial appendage. An additional objective was to investigate the long-term healing response of the atria and appendages after occlusion over a 3 month follow-up period.

## Materials and Methods

This study was performed in strict accordance with the recommendations of the Guide for the Care and Use of Laboratory Animals of the National Institutes of Health. The protocol was approved by the Committee on the Ethics of Animal Experiments of the Medical University of Gdańsk (Permit Number: 42/2013) and for experiments provided in Warmia and Mazury University, Faculty of Veterinary Medicine the protocol was approved by Local Ethic Committee for Animal Experiments in Olsztyn (Permit Number: 21/S/2013). All efforts were made to minimize the suffering of the animals. Protocols of anaesthesia and animal sacrifice used in experiment were consistent with standardised procedure of the Animal Laboratory established by Warmia and Mazury Univeristy.

### Device

**Fig 1:**
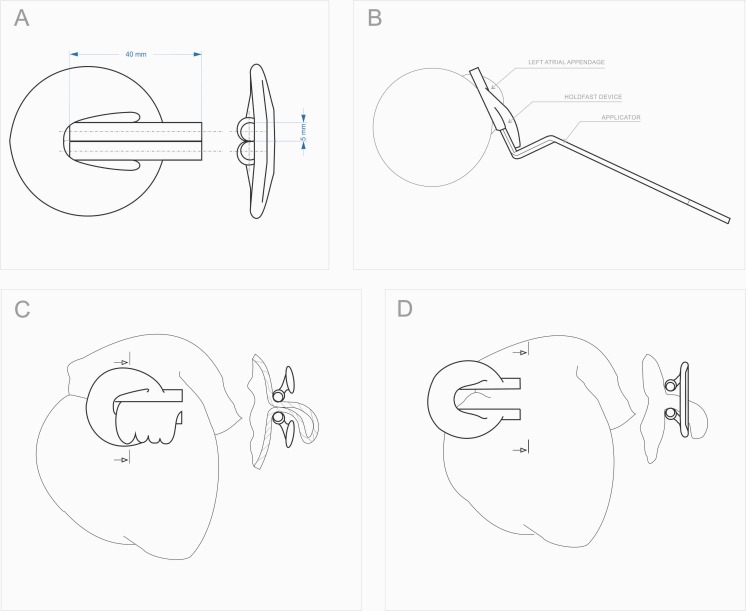
A. Clamp construction. The device structure and its dimensions, B. Installation of the device. The mounting of the clamp is accomplished with the use of specially designed surgical forceps. The short working arms of the forceps are design to enter the inside of the clamp tubes. By applying force to the long arm of the forceps the clamp opens and can be applied on the LAA, C. Device clamped on the LAA. The outline of the heart with a clamp on the left atrial appendage, D. Clamp in the maximum extension position. The outline of the heart with a clamp prior to the installation on the left atrial appendage.

For LAA occlusion, a special clamp was developed that consists of two tubes connected with an elastic bow (Fig 1). The tubes were specially constructed to create constant pressure on the base of the LAA and, therefore, to stop blood outflow (Fig 1). The device was monolithically made of polyamide powder PA2200 on a 3D printer using selective laser sintering technology. The biocompatibility of the material was certified according to ISO 10993–1.[14] The dimensions of the clip were calculated, designed and described by computer modelling (finite element method) so that after it is stretched to the thickness of a human LAA, approximately 6 mm, it will provide a clamping force of 36 N. This clamping force was found to be advantageous in the occlusion of LAA in large dogs.[2] The maximum extension of the tubes was set to a width of 10 mm. (Fig 1). This dimension was limited by the specially designed applicator. The device with the positioning forceps used for mounting is shown in Fig 1.

The construction of the clip is the subject of Patent Cooperation Treaty, registered under No. PCT / PL2014 / 000031.

### Study design

Fifteen Great White Polish pigs, seven males and eight females, at the age of 17 weeks were used to conduct the experiment. The animals used in experiments are not endangered and are not under the protection of Polish Law. The weight of the animals ranged from 55 to 60 kg, with an average of 57.93 kg. The animals were obtained from a single farm, and after arrival at the clinic, they were quarantined for 14 days to limit the impact of the environmental changes, living conditions, nutrition and transport stress on the general condition of the animals.

The study was designed to estimate the safety and feasibility of this novel procedure with an observation period of 14 days. Additionally, long-term follow up for efficacy and evaluation of the late inflammatory and tissue response was conducted for 90 days in 5 animals. In one swine of those five, the observation period was prolonged to 6 months to assess the long-term safety and histopathology of tissue ingrowth.

### Animals

The pigs were housed under consistent living conditions (day/night cycle of 12 h at 25 C) at the Central Animal Laboratory–Research and Service Centre of Warmia and Mazury University during the entire study. They were under the care of a veterinarian and had constant access to water and food.

All animals were starved for 12 hours before the surgery. Then, they were given the premedications of atropine at a dose of 0.05 mg / kg subcutaneously and azaperone (Stresnil) at a dose of 2.5 mg / kg intramuscularly. The induction of general anaesthesia was performed in day time mode using propofol (Scanofol) at an initial dosage of 5.0 mg / kg iv and was followed by intubation. Then, inhalation anaesthesia with 1–3% isoflurane (Aerane) with oxygen in a semi-closed system was administered continuously. During the operation, Ringer's solution was applied. In all cases, butorphanol (Butomidor) was administered intravenously at a dose of 0.2 mg / kg.

### Treatment model

Access to the mediastinum was obtained via left thoracotomy. The chest was opened at the fourth intercostal space. After reaching the pericardium, the left atrial appendage was located. The pericardial sac was opened along the long axis of the heart. The occlusion device was prepared and introduced. If necessary, the clip was repositioned using the specially designed applicator.

In all cases, the clip protruded beyond the pericardial sac. No attempt was made to close the pericardium. A drain was left in the thoracic cavity. Intercostal sutures were used to close the chest, followed by suture placement in the subcutaneous tissue and skin. After surgery, all animals were released into the care of a technician until they had fully recovered.

A single intramuscular injection of the antibiotics procaine benzylpenicillin and benzathine benzylpenicillin at 1 ml / 25 kg was performed during anaesthesia introduction. For five consecutive days, metamizol pain therapy (Biovetalgin) was administered at a dose of 30 mg / kg IM.

The animals were euthanized at the end of each time point for the heart explantation and post-mortem examination.

The initial observation period was fourteen days. At that time point, 10 pigs were euthanized. Four animals were observed for a total of 3 months, and one animal was observed for a total of 6 months. At death, the hearts were explanted from all animals by thoracotomy.

### Histopathological examination

The hearts were delivered in 10% formalin solution to the Department of Pathology, Medical University of Gdansk. Macroscopic and microscopic evaluation of left atrial appendage occlusion was performed at the point of clip pressure. Histological tests were performed on excised samples of 5x5 mm prepared according to the standard pathological procedure. First they were expanded in 10% formalin for approximately 48 hours. Then, they were dehydrated with a series of appropriate concentrations of alcohol and xylene, and finally, they were embedded in low melting paraffin. Each paraffin block was cut into 4 mm sections. All samples contained a piece of the clip and LAA. The material was then prepared for the histological tests with hematoxylin and eosin (H & E).

### Statistical analysis

No specific statistical analysis was needed. Continuous variables were expressed as mean values. Cathegorical variables were expressed as percentage.

## Results

There were no perioperative deaths. In three subjects (20%), ventricular arrhythmia occurred during the open heart procedure and was resolved after discontinuation of the manipulation. After intravenous administration of 80 mg lignocaine, no additional arrhythmias were observed throughout the rest of the procedure. In one animal (6.66%), bradycardia occurred, and it subsided after discontinuation of the manipulation.

After surgery, all animals fully awakened and normal physiological activity was observed.

At all time points, the hearts were evaluated macro- and microscopically to assess the tissue inflammatory response to the polyamide powder and the adhesion formation between the LAA walls at the clamp site.

The macroscopic assessment of the left atrium revealed no clots in the area of the appendage. Furthermore, the surface of the wall was smooth and shiny, and at the clamp site, there was constant firm compression of the tubes on the LAA wall. Full adherence of the walls in the line of the tubes was achieved without complete concrescence or signs of atrophy of the LAA. The external surface of the LAA and the area around the clamp were covered with a thin layer of translucent fibrous connective tissue.

**Fig 2:**
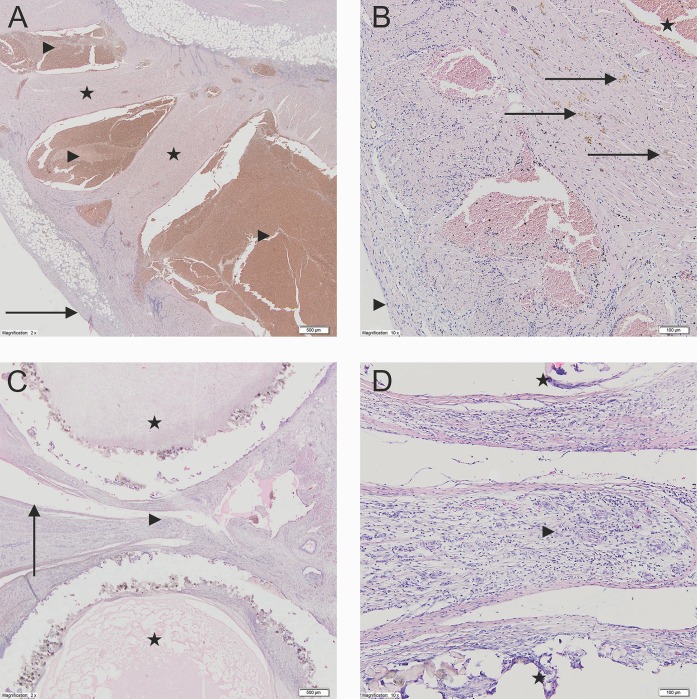
A—The LAA interior filled with organized blood clots. Left atrial appendage (2x). The LAA interior site filled with organized blood clots. The myocardium is present with chronic nonspecific inflammatory infiltration. The LAA surface is present with newly forming granulation tissue with dispersed fibroblasts (*Stars*–the muscle tissue of the left atrial appendage; *Arrowhead*—The LAA interior site; *Arrow*–the surface of left atrial appendage). B—New granulation tissue with dispersed fibroblasts. Left atrial appendage (10x). In between the cardiomyocytes of the left atrial appendage there are diffused fibroblast, hemosiderophags and chronic nonspecific inflammatory infiltration cells (*Stars*—The LAA interior site; *Arrows*–hemosiderophags; *Arrowhead*–the outer surface of the left atrial appendage). C—Single layer of endothelial cells between and around the tubes. The cross-section through the clamp (2x) Single layer of endothelial cells between the compressed tubes. Between and around the clamps the fibrous connective tissue is visible. Around the clamp elements the creation of the foreign body type granulomas are not observed (*Stars*–clamp tubes; *Arrowheads*—fibrous connective tissue in between the tubes; *Arrow*–The clam site surface of the LAA wall). D—Mature granulation tissue and elements of chronic inflammatory infiltration. The cross-section through the clamp (10x). In between the tubes the creation of the LAA walls adhesion is visible with the formation of mature granulation tissue and the appearance of fibroblast and lymphoid chronic inflammatory infiltration cells (*Stars*–clamp tubes; *Arrowhead*–mature fibrous connective tissue in between the tubes).

**Fig 3:**
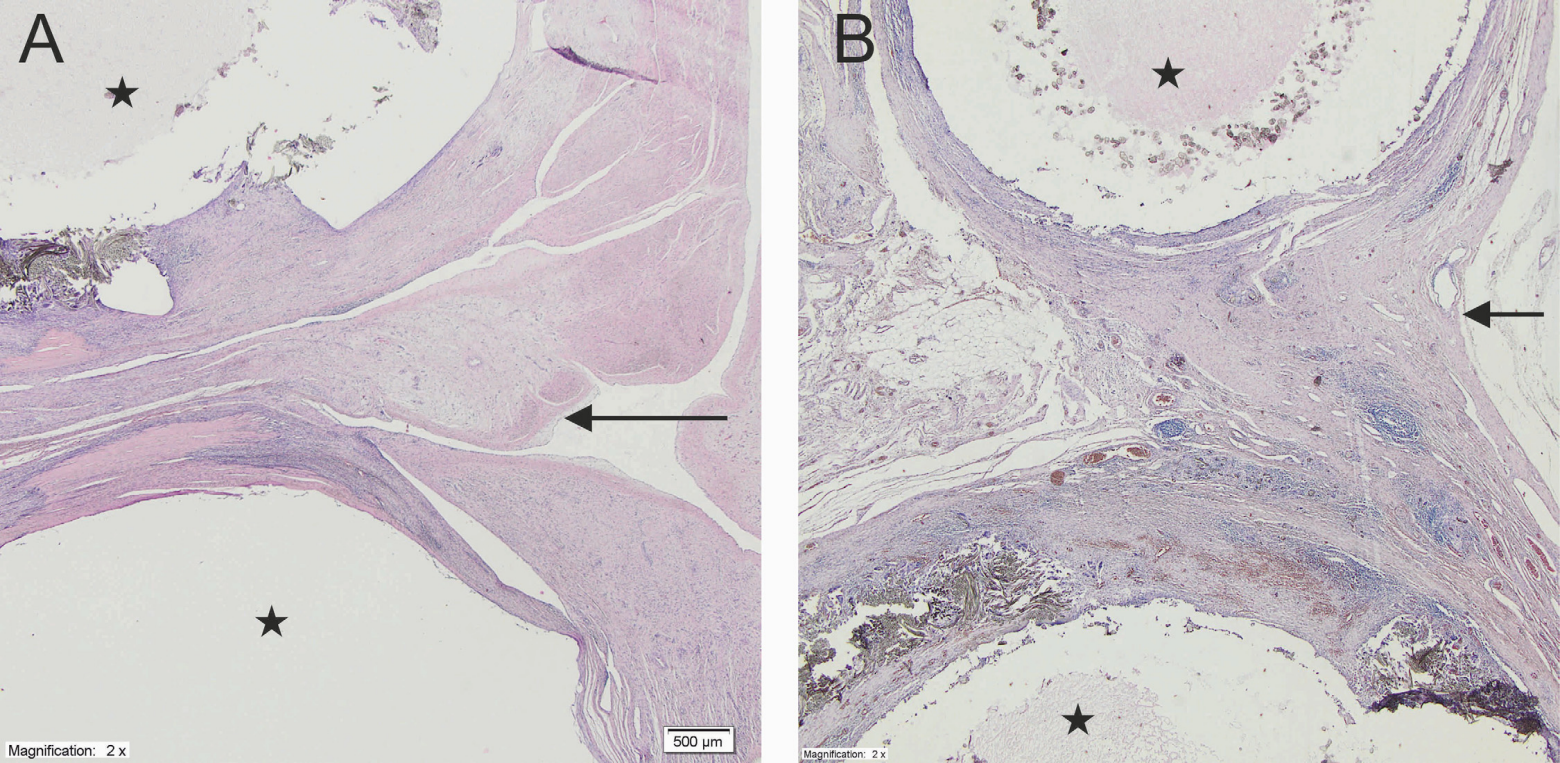
**Histologic findings of the cross section of the occlusion site at 90 days (A) at 6 months (B) (x2).** At both time points fibrous connective tissue with fibroblast and chronic inflammatory infiltrate was found between both fabrics and around the fabrics. At 6 months’ tissue contains more elastic fibres and lymphocytic infiltrate was less prominent. Left atrial appendage was covered by one layer of endothelial cells (*Stars*–clump; *Arrow*—left atrial appendage).

Histologically, the distal portion of the LAA was mainly built of myocardiocytes with numerous hemosiderophages between them. The LAA interior was filled with organized blood clots (Fig 2), and the surface had newly forming granulation tissue with dispersed fibroblasts (Fig 2). The surface area of the atrium next to the clip was covered with a single layer of endothelial cells (Fig 2), and between and around the tubes, mature granulation tissue and elements of chronic inflammatory infiltration were observed (Fig 2). Focally, granulation tissue had been replaced by fibrous connective tissue. Histopathological changes in the left atrium for time points at 90 days and 6 months are shown on Fig 3.

## Discussion

A variety of techniques and devices for ligation, excision, or exclusion of the LAA, as adjuncts to cardiac operations and as a sole therapy, have been described in the literature. Currently there are many devices for closure of the left atrial appendage. Methods include closure from the inside (via percutaneous intervention) and closure by surgical procedures, including sternotomy and less invasive methods.[15]

Studies have shown that exclusion of the LAA during mitral valve operations reduced the risk of subsequent stroke.[11] Surgical epicardial occlusion of the LAA was evaluated in the Left Atrial Appendage Occlusion Study (LAAOS) trial[16] in patients undergoing coronary artery bypass grafting with an increased risk of stroke (CHADS score > 2), and a 12% rate of LAA injury requiring further suture repair was reported. Predischarge studies showed that the complete appendage closure rates were only 45% using sutures and 75% using staples.[17] Incomplete closure was associated with an increased rate of thromboembolic events.[18]

In a retrospective study of patients with exclusion or excision of the LAA, complete closure of the LAA was only successful in 40% of patients. Successful closure occurred more often with excision of the LAA (73%) than with suture exclusion (23%) or stapler exclusion (0%).[8] Of the patients for whom exclusion was unsuccessful, persistent flow into the appendage was found in 100% of the staple exclusions and in 50% of the suture exclusions. The prevalence of thrombus in the appendages with persistent flow was high: 46% with suture exclusion and 67% with staple exclusion.[12]

Percutaneous endocardial devices have been evaluated and they have shown decreased stroke rates compared with controls. However, strokes can not be completely eliminated by percutaneous endocardial devices, and echocardiographic studies have shown residual flow into the appendage in some cases.[12] There have been episodes of device dislodgement[17], and when this situation occurs, the device will cause a foreign body reaction in contact with the blood and requires oral anticoagulation treatment.

External application of a flexible band has been shown to be effective in LAA exclusion in experimental studies.[12] Titanium and nitinol clip covered with knitted Dacron (DuPont, Wilmington, DE) was effective in a clinical trial of 34 patients with no evidence of leakage or any hemodynamic consequences.[13] Those studies showed that an externally placed device is adaptable to the variable anatomy of the LAA.

For percutaneous intravascular approaches, the first device developed was the PLAATO System. It consists of a nitinol self-expanding cage covered with a polytetrafluoroethylene membrane.[19] Although that device was not developed further, it led to the implementation of a minimally invasive approach for preventing strokes, closure of the left cardiac auricle.

The Watchman (Boston Scientific, Natick, MA) is a nitinol self-expanding cage covered with fabric. The cage expands the cavity of the left cardiac auricle. The device is inserted directly into the left auricle using a 14F access sheath. A slight flow in the auricle after application was observed in approximately 41% of patients.[20] The Amplatzer Cardiac Plug (St. Jude Medical, Minneapolis, MN) is a nitinol self-expanding cage consisting of two disks that each have a polyurethane patch. It is applied through a femoral vein and puncture of the interatrial septum. The device closes the orifice of the left cardiac auricle.[21] The LARIAT device (SentreHeart, Redwood City, CA) enables intravascular ligation of the auricle by connecting the transseptal access to the caudal side of the xhiphoid process. The application of this device requires computer tomography to evaluate anatomic ratios. It also has many limitations, e.g., the size of auricle must be greater than 40 mm.[22]

The application of an endovascular device can cause many different complications associated with mortality risk, such as bleeding in the access place, pericardial exudate which can result in tamponade and air embolisms.[23]

During cardiac surgical procedures, suturing of the auricle can be replaced with the application of a closure device, such as the AtriClip (Atricure, West Chester, OH) which has been already widely described. [2,24] Another device enabling closure of the auricle during cardiac surgical procedures is the Tigerpaw System (Maquet).[25]

The goal of this study was to develop and evaluate a new occlusion tool for isolating the atrial appendage. The device has a few advantages that have to be mentioned. First of all, the design enables clip implantation as a stand-alone procedure via mini-thoracotom. The specially designed deployment system allows its proper placement without the necessity for adhesiolisis, furthermore the system also provides the possibility of repositioning of the clamp after the initial insertion contrary to other external and internal LAA occluding devices. With the Use of additive manufacturing (laser sintering technology) we obtain a monolithical product with reproducible physio-mechanical properties in many sizes. Hence it has a potential to be used as personalized treatment.

Importantly, there were no major complications associated with the deployment of the occluder, and the device did not migrate over the observation period. The clamp designed and proposed by the researchers of this study was associated with mild adhesions and a mild inflammatory response; however, neither pericarditis nor other medical complications related to tissue reactions to the implant were observed. The most important finding of the present study was that the implantation of the clip resulted in 100% exclusion of the LAA from the rest of the atria. The success of sealing was confirmed by physical and histopathological examination.

Post-mortem examination showed that the tissue around the device had fully healed with proper macroscopic scar formation. Histopathology showed a fibrous scar on one side of the atrium and proper endothelial cells inside the atrium.

A limitation of the present study is that the occlusive devices were applied to normal atria in healthy pigs. Human candidates for surgical atrial exclusion often have enlarged left atria[8] and might require a larger device than those used in present study. Furthermore, the appendages of older patients with various comorbidities might be more prone to tearing than those of the relatively young and healthy pigs used in the present study.

Another drawback of this device is its relatively large size; however, this characteristic did not influence the positioning or ease of implantation.

Both the number of pigs and the follow up period were consistent with the European EN ISO 10993–6:2009 Norm (Biological evaluation of medical devices–Part 6: Tests for local effects after implantation).

Because of the ease of application, minimal morbidity, and high level of effective occlusion of the LAA, placement of an external occlusion device may become standard in elective cardiac operations to prevent LAA thrombus, particularly as the human population ages and the incidence of atrial fibrillation increases.

## Conclusion

In conclusion, our study demonstrated the safety and efficacy of this novel LAA exclusion device, with rapid closure times, reduced rates of bleeding and tissue tearing and proper fibrous scar formation. The results suggest that this new occlusion device may be an equivalent or superior alternative to manual mattress suturing or stapling with or without reinforcement. Future studies will investigate the proposed occlusion method on a larger sample with a more representative LAA size of that observed in patients that are candidates for surgical atrial exclusion. A multicentre phase II clinical trial should be performed to demonstrate successful occlusion in human subjects using this device.

## Supporting Information

S1 File The ARRIVE Guidelines Checklist. (PDF)
